# Comparison of two Turnip mosaic virus P1 proteins in their ability to co-localize with the *Arabidopsis thaliana* G3BP-2 protein

**DOI:** 10.1007/s11262-021-01829-w

**Published:** 2021-02-18

**Authors:** Hendrik Reuper, Björn Krenz

**Affiliations:** Leibniz Institute DSMZ-German Collection of Microorganisms and Cell Cultures, Inhoffenstr. 7 B, 38124 Braunschweig, Germany

**Keywords:** Potyvirus, Turnip mosaic virus, P1 protein, G3BP, Stress granules

## Abstract

Turnip mosaic virus (TuMV), belonging to the genus Potyvirus (family *Potyviridae*), has a large host range and consists of a single-stranded positive sense RNA genome encoding 12 proteins, including the P1 protease. This protein which is separated from the polyprotein by cis cleavage at its respective C-terminus, has been attributed with different functions during potyviral infection of plants. P1 of Turnip mosaic virus (P1-TuMV) harbors an FGSF-motif and FGSL-motif at its N-terminus. This motif is predicted to be a binding site for the host Ras GTPase-activating protein-binding protein (G3BP), which is a key factor for stress granule (SG) formation in mammalian systems and often targeted by viruses to inhibit SG formation. We therefore hypothesized that P1-TuMV might interact with G3BP to control and regulate plant SGs to optimize cellular conditions for the production of viral proteins. Here, we analyzed the co-localization of the *Arabidopsis thaliana* G3BP-2 with the P1 of two TuMV isolates, namely UK 1 and DEU 2. Surprisingly, P1-TuMV-DEU 2 co-localized with AtG3BP-2 under abiotic stress conditions, whereas P1-TuMV-UK 1 did not. AtG3BP-2::RFP showed strong SGs formation after stress, while P1-UK 1::eGFP maintained a chloroplastic signal under stress conditions, the signal of P1-DEU 2::eGFP co-localized with that of AtG3BP-2::RFP. This indicates a specific interaction between P1-DEU 2 and the AtG3BP family which is not solely based on the canonical interaction motifs.

**Supplementary Information:**

The online version of this article (10.1007/s11262-021-01829-w) contains supplementary material, which is available to authorized users.

## Introduction

The *Potyviridae*, members of which have single-stranded, positive sense RNA genomes, is the largest family of RNA plant viruses, and includes many agriculturally important viruses. The typical potyviral genome contains one long open reading frame (ORF) and another relatively short ORF resulting from transcriptional slippage [1]. The resulting two polyproteins are ultimately processed into 12 mature proteins by three viral proteases [2, 3]; and references therein]. The P1 protein, localized at the N-terminus (N-ter) of the potyviral polyprotein, is a serine endopeptidase. It is self-released by cis cleavage at its C-terminus [4], thereby preventing early host defense responses, that would be detrimental to virus systemic infection [5]. P1 contains a broad specific RNA binding domain, spanning residues 150 to 168, the properties of which have been experimentally confirmed [6]. P1 is the most variable potyviral protein in terms of sequence length and amino acid composition and its involvement in the adaptive process and host range specificity has been previously reported [7]. The high sequence variability of P1 associated with a high degree of structural disorder strongly supports the hypothesis of mutational robustness and the lower-evolutionary constraint effect related to disorder; a feature already observed in various proteins [8]. This could be related to a higher adaptability to various environments, a disorder-based feature already discussed for RNA-viruses [8]. In this context, P1 has already been linked to host range specificity within the *Potyviridae* family reinforcing the idea that disorder could act as an enhancer of adaptation [8].

Stress granules are cytoplasmic mRNA-protein-complexes which assemble in response to biotic (e.g. viral or microbial infection) or abiotic (e.g. high salt or drought conditions) stress to halt translation and enable translational stress response (reviewed in [9]). The Ras-GAP SH3 domain–binding protein (G3BP) has been shown to be key factor of SG formation in mammalian systems. Due to their ability to halt translation, and the high density of polysomal components, SGs in general and G3BP in particular are a major target of viral proteins [10–12]. The formation of SGs can be inhibited by viruses in various ways; for example, the C-terminal domain of nonstructural protein 3 (nsP3) of Semliki Forest virus (SFV) can bind and sequester human G3BP (HsG3BP) into viral RNA replication complexes. The binding domain of nsP3 to HsG3BP has been mapped to two tandem ‘FGDF’ repeat motifs close to the C-terminus of the viral protein. Not only can ‘FGDF’-like G3BP-binding motifs be found in different plant virus proteins but also G3BP homologs are present in many plant species [13, 14]. The P1 protease of Turnip mosaic virus (TuMV) and the nuclear shuttle protein (NSP) of different begomoviruses, for example Abutilon mosaic virus (AbMV) and the Cabbage leaf curl virus (CabLCV), harbor ‘FGDF’-like motifs. Recently it was shown that the last 31 amino acids at C-terminus of the NSP of AbMV, which harbors the ‘FGDF’-like motif, co-localized and interacted with a G3BP from *Arabidopsis thaliana* (AtG3BP-2) [15].

Here, we studied the ability of the AtG3BP-2 and AtG3BP-4, another potential member of the *A. thaliana* G3BP family, to co-localize with the P1 proteases of two different TuMV isolates, namely UK 1 and DEU 2. The isolate UK 1 was isolated from *Brassica napus* in the United Kingdom in 1975 while DEU 2 was isolated from *Raphanus sativus* in Germany [16].

## Results and Discussion

**Fig. 1:**
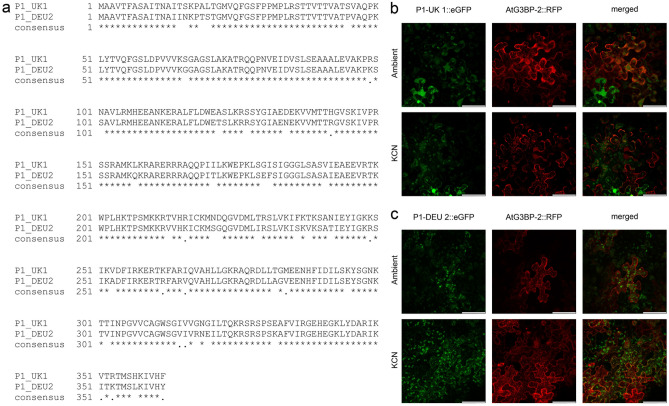
P1 amino acid sequences from the isolates UK 1 and DEU 2 were aligned and conserved residues were highlighted. Both ORFs and AtG3BP-2 (without stop codon) were amplified by PCR and inserted into the expression vectors pK7FWG2 and pGWB454, respectively. Both plasmids express the GOI under the control of a CaMV 35S promoter. The vectors were transformed into *Agrobacterium tumefaciens* and transiently expressed in *N. benthamiana* leaves. Epithelial cells were imaged at 2 dpi by confocal laser-scanning microscopy using a Leica TCS SP8 confocal laser-scanning microscope. **a** Amino acid sequence alignment of P1-UK 1 and P1-DEU 2. **b** P1-UK 1::eGFP co-expressed with AtG3BP-2::RFP under ambient conditions and KCN stress. P1-UK 1 is showing a nuclear-cytoplasmic signal as well as a chloroplast signal at both conditions while AtG3BP-2 forms more SGs under stress conditions. **c** P1-DEU 2::eGFP shows a mostly chloroplast signal at ambient conditions but co-localizes with AtG3BP-2::RFP after KCN application. All images are z-stack maximum projections and correspond to a size of 185 µm × 185 µm, the scale bar is 50 µm

The open reading frames of the P1 proteins from two different TuMV isolates (UK 1: NC_002509; DEU 2: AB701700) as well as AtG3BP-2 (AT5G43960) and AtG3BP-4 (AT1G69250) were amplified by PCR and inserted into the vector pENTR™/D-TOPO™ (Invitrogen). For plant expression, the fragments were subsequently recombined into the Gateway ® compatible destination vectors pK7FWG2 (C-terminal eGFP) [17] and pGWB454 (C-terminal mRFP) [18] using L/R-Clonase™ II enzyme mix (Invitrogen) and transformed into *Agrobacterium tumefaciens* strain LBA4404. P1-UK 1::eGFP and P1-DEU 2::eGFP fusion proteins were transiently co-expressed with AtG3BP-2::mRFP in *Nicotiana benthamiana* epidermal cells and cellular localization was studied under ambient conditions and oxidative stress (KCN) 2 days post infiltration (dpi) by confocal laser-scanning microscopy (Leica TCS SP8). Experiments in the mammalian system have shown the interaction of viral proteins with human G3BP via an ‘FGDF’ motif [19]. As the P1 protease of TuMV contains two ‘FGDF’-like motifs, (‘FGSF’ at amino acid position 26–29 and ‘FGSL’ at 56–59) we hypothesized that P1-TuMV could bind to AtG3BP-2 as well. We compared two P1 proteases from different isolates of TuMV, UK 1 and DEU 2, sharing 96.4% amino acid sequence similarity (Fig. 1a). The two isolates were obtained from different host plants, namely *B. napus* (UK 1) and *R. sativus* (DEU 2), but they both belong to the world-B group [16]. Both P1 constructs showed a chloroplast signal under ambient conditions while AtG3BP-2 showed a mostly cytoplasmic signal with a few granular structures. The formation of SGs under ambient conditions by G3BP overexpression alone in absence of other stresses has been reported previously [20]. After KCN treatment (2 mM, 20 min; [15]), to induce abiotic stress, AtG3BP-2::RFP showed strong SG formation. While P1-UK 1::eGFP maintained a chloroplastic signal under stress conditions, the signal of P1-DEU 2::eGFP also shifted into granular structures whose signals co-localized with that of AtG3BP-2::RFP (Fig. 1b, c). P1-DEU 2::eGFP also co-localized with AtG3BP-4::RFP after KCN treatment, while P1-UK 1::eGFP showed no such interaction (Fig. S1). The similar interaction pattern of AtG3BP-2 and -4 with the different P1 variants could be considered an indication of a potential level of redundancy among the individual proteins within the AtG3BP family. The P1::eGFP constructs were also infiltrated alone and treated with KCN to induce stress conditions. In absence of AtG3BP-2::RFP, both P1::eGFP constructs retained their chloroplastic localization at ambient conditions and under KCN stress (Fig. S2). Furthermore, the behavior of the two P1 constructs in the background of a viral infection was investigated. For this purpose, the P1 constructs were co-infiltrated with AtG3BP-2 into *N.* *benthamiana* leaves and these were rub-inoculated along with either of the two virus isolates, respectively. TuMV UK 1 also expresses a RFP with a nuclear localization signal (NLS) [21], to highlight infected cells. Images were taken 3 dpi. However, SG formation and associated co-localization of P1 and AtG3BP-2 could not be observed in infected cells. SG dispersion or the prevention of their formation has been previously described not only for mammalian viruses but also for plant viruses, such as potato virus X [22–24] and seems also to be the case for TuMV (Fig. S3). For P1-UK 1 ambient and -DEU 2 KCN in background with TuMV UK 1-RFP::NLS, it can be seen that SG can be formed in uninfected cells that do not show an RFP signal in the nucleus simultaneously. Directly adjacent cells, which show a cytoplasmic RFP signal from AtG3BP-2 in addition to the nuclear RFP signal of TuMV UK 1-RFP::NLS, no SG formation is detectable even under KCN stress. This also applies to the samples inoculated with TuMV DEU 2. These findings indicate a specific interaction between P1-DEU 2 and members of the *A. thaliana* G3BP family which is not solely based on the canonical interaction motifs but also on factors or motifs which yet remain to be elucidated. It is suspected that acidic residues downstream of the binding motif may also play a role in the actual binding capability (reviewed in[13]). However, both proteins contain an aspartic acid directly downstream of the second binding motif. The only difference is at residue 66 where P1-UK 1 harbors a serine and P1-DEU 2 a glycine. So far, neither of these amino acids has been linked to the interaction between viral proteins and G3BP. Particular focus in the future should therefore be on the role of these residues in mediating a putative interaction between P1 and AtG3BP-2 (and other AtG3BPs). Analysis of mutants with substituted residues will shed more light on the role of P1 in the onset of early viral infection and SG dispersion. Other studies have already investigated the properties of TuMV proteins from *B. napus* and *R. sativus* isolates and found differences in intracellular localization and their interaction partners. Lopez-Gonzales et al. compared the P3 protein of isolates UK 1 and JPN 1, like DEU 2 isolated from *R. sativus*, and found that they behaved differently towards 6K2, another TuMV protein [25]. Thus, P3 UK 1 co-localized with 6K2 in ER-derived 6K2-induced vehicles while this co-localization was not observed for P3 JPN 1. Based on these results, the authors propose that single amino acids exchanges in the proteins of the two isolates are responsible for the different viral symptoms and the sub-cellular localization of the P3 protein.

Further research will help to elucidate the interplay between plant viruses and their respective host and the importance of SGs as pro- or anti-viral factors. For this purpose, the infection efficiency of the various TuMV isolates in different host plants, e.g. *A. thaliana* and *N. benthamiana*, will be investigated. Furthermore, infection experiments in G3BP-KO and overexpression lines will help to better understand the role of TuMV-P1 and SGs during the onset and progression of a viral infection.

## Electronic supplementary material

Below is the link to the electronic supplementary material.

Electronic supplementary material 1 (PDF 1465 kb)
