# Supplementary material for: Comparison of two Turnip mosaic virus P1 proteins in their ability to co-localize with the Arabidopsis thaliana G3BP-2 protein
Source: Virus Genes. 2021 Feb 18;57(2):233–7. doi: 10.1007/s11262-021-01829-w (PMC7985126; doi:10.1007/s11262-021-01829-w)
Supplement: Supplementary file 1 — Electronic supplementary material 1 (PDF 1465 kb) [file 11262_2021_1829_MOESM1_ESM.pdf]

Short communication in Virus Genes

Comparison of two Turnip mosaic virus P1 proteins in their ability to co-localize with the *Arabidopsis thaliana* G3BP-2 protein

Hendrik Reuper and Björn Krenz*

Leibniz Institute DSMZ-German Collection of Microorganisms and Cell Cultures, Inhoffenstr. 7 B, 38124 Braunschweig, Germany.

* Correspondence: bjoern.krenz@dsmz.de; Tel.: +49-531-2616-407; Fax.: +49-531-2616-418

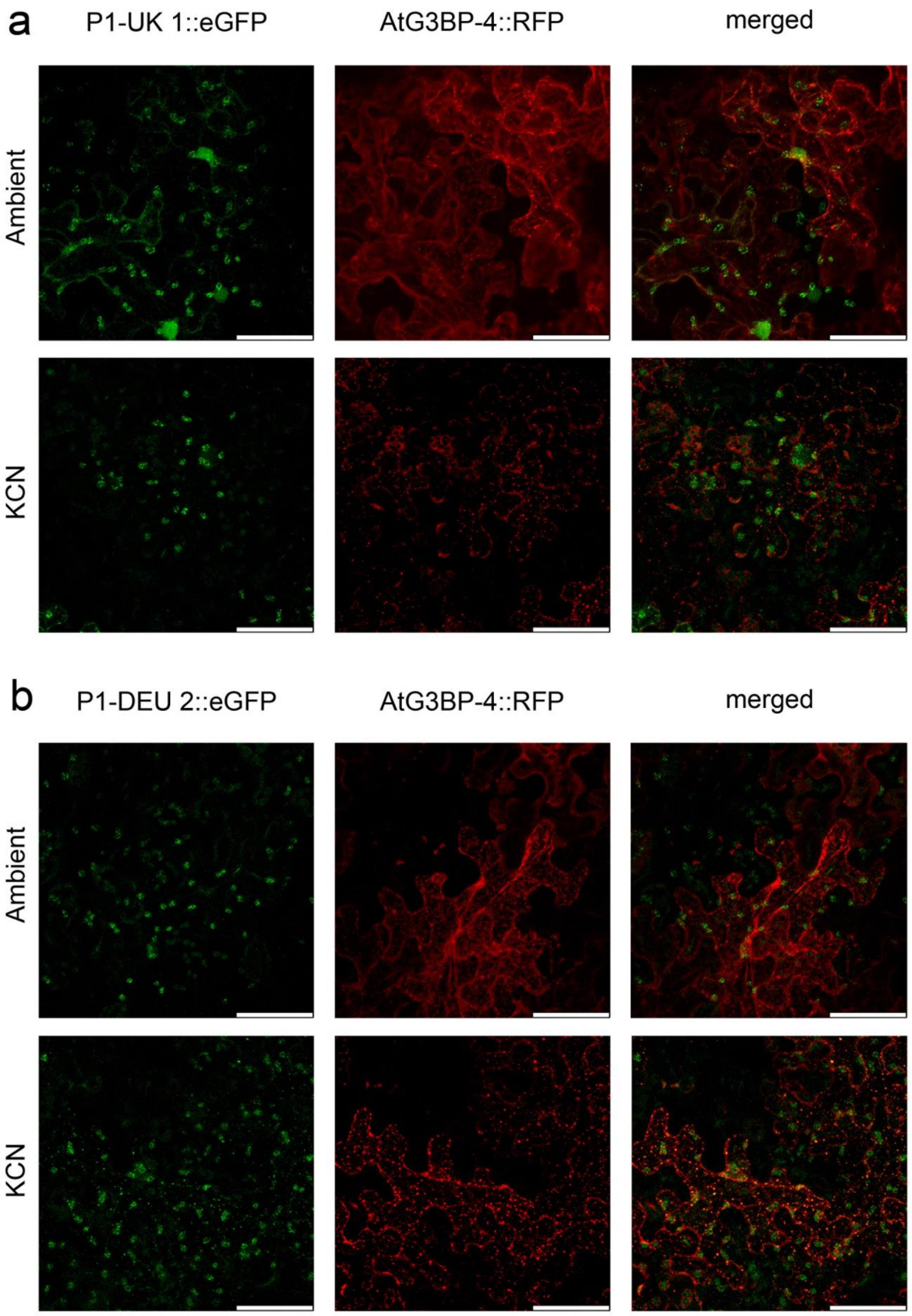

Fig. S1 TuMV-P1 interaction with AtG3BP-4 a) Co-expression of P1-UK 1::eGFP with AtG3BP-4::RFP in *N. benthamiana* leaves under ambient conditions and after KCN treatment. P1-UK 1 signal is mostly chloroplastic with a weaker nuclear-cytoplasmic signal at ambient conditions which shifts completely

to chloroplasts under KCN stress. AtG3BP-4 locates mostly to the cytoplasm under ambient conditions and forms more SGs under KCN stress conditions. b) P1-DEU 2::eGFP shows a strong chloroplast signal under both conditions but co-localizes with AtG3BP-4::RFP after KCN application. The images are maximum projections of z-stacks and correspond to a size of 185 µm x 185 µm. The scale bar is 50 µm

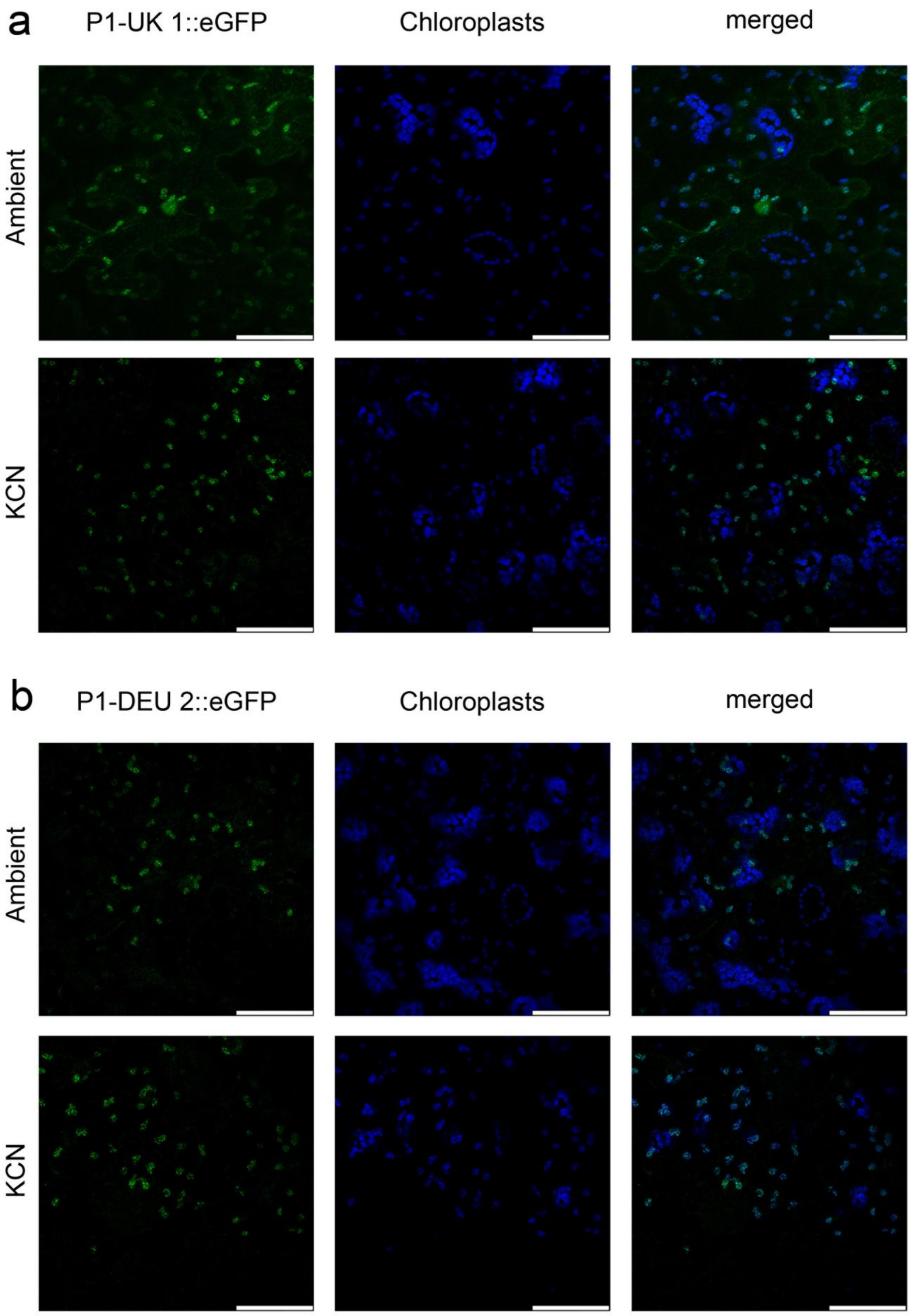

Fig. S2 Localization of TuMV-P1::eGFP constructs in *N. benthamiana* leaves a) Co-localization of P1-UK 1::eGFP with chloroplasts (pictured in blue) under ambient und KCN stress conditions. b) P1-DEU 2::eGFP also shows a chloroplastic signal which does not alter under KCN stress. Pictures were

obtained 2 dpi and the scale bar corresponds to 50 µm. All pictures are maximum projections of z-stacks

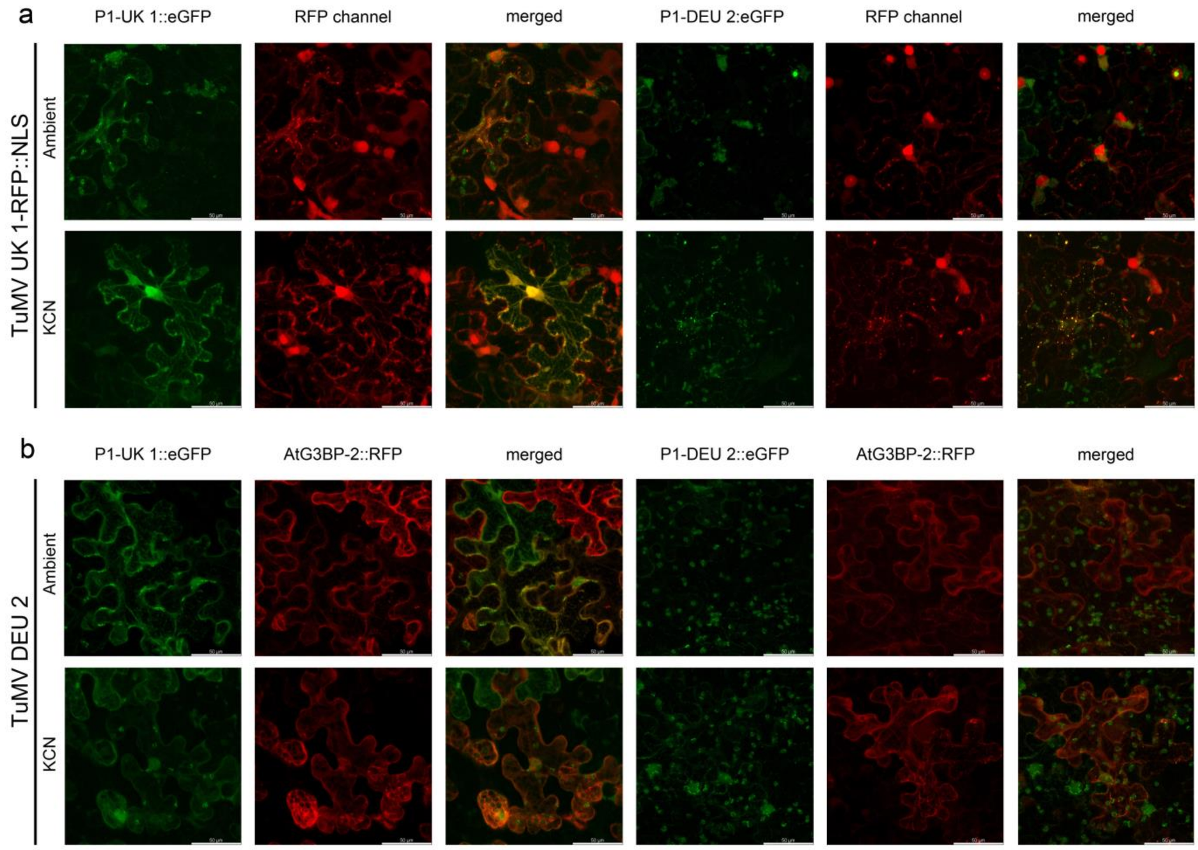

Fig. S3 Localization of potyviral P1::eGFP constructs in the background of a local viral infection. a) Co-expression of potyviral P1::eGFP constructs as indicated with AtG3BP-2::RFP in TuMV UK 1-RFP::NLS [21] infected *N. benthamiana* epidermis cells under ambient and KCN-stress conditions. TuMV UK 1-RFP::NLS -infected cells can be recognized by the RFP signal in the nucleus. b) Cells expressing the P1::eGFPs together with AtG3BP-2::RFP in the background of a local infection with TuMV DEU 2. Note that the TuMV DEU 2 does not express a fluorescent marker protein to visualize infected cells. Noteworthy, no SGs formation or co-localization of P1 and AtG3BP-2 could be observed. Infiltrated leaves were inoculated by mechanical inoculation with crude sap from infected *N. benthamiana* leaves and pictures were obtained 3 dpi. Images were obtained 3 dpi and are maximum projections of z-stacks. The scale bar represents 50 µm.
